# A proof-of-concept study on mortality prediction with machine learning algorithms using burn intensive care data

**DOI:** 10.1177/20595131211066585

**Published:** 2022-02-18

**Authors:** Jian Fransén, Johan Lundin, Filip Fredén, Fredrik Huss

**Affiliations:** 1 Department of Surgical Sciences, Plastic Surgery, 8097 Uppsala University, Uppsala, Sweden; 2 123913 Karolinska Institute Department of Global Public Health, Stockholm, Sweden; 3 FIMM, Institute for Molecular Medicine, Helsinki, Finland; 4 Department of Anaesthesia and Intensive Care, 59561 Uppsala University Hospital, Uppsala, Sweden; 5 Department of Plastic- and Maxillofacial Surgery, 59561 Uppsala University Hospital, Uppsala, Sweden

**Keywords:** machine learning, mortality prediction, burn care, intensive care, clinical databases, computer-based prediction

## Abstract

**Introduction:**

Burn injuries are a common traumatic injury. Large burns have high mortality requiring intensive care and accurate mortality predictions. To assess if machine learning (ML) could improve predictions, ML algorithms were tested and compared with the original and revised Baux score.

**Methods:**

Admission data and mortality outcomes were collected from patients at Uppsala University Hospital Burn Centre from 2002 to 2019. Prognostic variables were selected, ML algorithms trained and predictions assessed by analysis of the area under the receiver operating characteristic curve (AUC). Comparison was made with Baux scores using DeLong test

**Results:**

A total of 17 prognostic variables were selected from 92 patients. AUCs in leave-one-out cross-validation for a decision tree model, an extreme boosting model, a random forest model, a support-vector machine (SVM) model and a generalised linear regression model (GLM) were 0.83 (95% confidence interval [CI] = 0.72–0.94), 0.92 (95% CI = 0.84–1), 0.92 (95% CI = 0.84–1), 0.92 (95% CI = 0.84–1) and 0.84 (95% CI = 0.74–0.94), respectively. AUCs for the Baux score and revised Baux score were 0.85 (95% CI = 0.75–0.95) and 0.84 (95% CI = 0.74–0.94). No significant differences were observed when comparing ML algorithms with Baux score and revised Baux score. Secondary variable selection was made to analyse model performance.

**Conclusion:**

This proof-of-concept study showed initial credibility in using ML algorithms to predict mortality in burn patients. The sample size was small and future studies are needed with larger sample sizes, further variable selections and prospective testing of the algorithms.

**Lay Summary:**

Burn injuries are one of the most common traumatic injuries especially in countries with limited prevention and healthcare resources. To treat a patient with large burns who has been admitted to an intensive care unit, it is often necessary to assess the risk of a fatal outcome. Physicians traditionally use simplified scores to calculate risks. One commonly used score, the Baux score, uses age of the patient and the size of the burn to predict the risk of death. Adding the factor of inhalation injury, the score is then called the revised Baux score. However, there are a number of additional causes that can influence the risk of fatal outcomes that Baux scores do not take into account. Machine learning is a method of data modelling where the system learns to predict outcomes based on previous cases and is a branch of artificial intelligence. In this study we evaluated several machine learning methods for outcome prediction in patients admitted for burn injury. We gathered data on 93 patients at admission to the intensive care unit and our experiments show that machine learning methods can reach an accuracy comparable with Baux scores in calculating the risk of fatal outcomes. This study represents a proof of principle and future studies on larger patient series are required to verify our results as well as to evaluate the methods on patients in real-life situations.

## Introduction

Burns were the fourth most common type of traumatic injury in 2004 worldwide and 11 million people were injured through burns,^1^ with an estimated mortality of 180.000 annually.^2^ In Sweden, approximately 36,000 people are treated for burns annually with around 1300 needing hospital care.^3^

The need for the prediction of burn outcome has led to the invention of several scoring systems. One of the common mortality prediction scores for burns is the Baux score or the revised Baux score. The Baux score is calculated by adding the total body surface area (TBSA) burned (as a percentage) to the patient's age. With revision, the presence of an inhalation injury adds another 17 points to the final score where higher scores indicate increased risk.^4,5^ The Baux score and the revised Baux score have been shown to be reliable,^6–9^ but it is suggested that more advanced statistical models could produce better predictions.^5^

Examples of other burn scores are the Abbreviated Burn Severity Index (ABSI), the Belgian Outcome of Burn Injury (BOBI), the Acute Physiology And Chronic Health Evaluation II (APACHE II)^7^ and the Boston score.^6^

Intensive care scores such as the National Early Warning Score (NEWS),^10^ the Sequential Organ Failure Assessment (SOFA)^11^ and the Simplified Acute Physiology Score III (SAPS III)^12^ are other more general prediction models frequently used at our burn centre.

Artificial intelligence (AI) and its sub-branch machine learning (ML) are rapidly emerging areas of research in medicine.^13^ A large number of ML models have been evaluated for predictive purposes, for example in cancer diagnostics,^14–18^ radiology^19,20^ and pathology.^21^ In contrast to static mathematical formulas, ML does not assume prior relationships but uses input data to ‘learn’ from previous data with known outcomes and then tests on new cases with outcomes removed. The likelihood of correctly predicted outcomes will determine the accuracy of the model.^22^

Types of ML models are many and each has different builds to optimise performance of the algorithm. In brief, visual representation of these mathematical algorithms can sometimes be described as decision trees or networks similar to neuronal matrices. Examples of models are random forest, decision tree, logistic regression and artificial neural networks.

ML has previously been studied in burn intensive care for the prediction of mortality and length of hospital stay (LOS) using intensive care unit (ICU) input data.^23–28^ Several studies have predicted mortality using different ML algorithms in comparison with outcome mortality presenting results in area under the curve (AUC) of receiver operating characteristic (ROC) curves. For example, Stylianou et al. predicted mortality at an accuracy of 0.945 for random forest, 0.967 for Support-vector machine, 0.974 for artificial neural network, 0.971 for logistic regression and 0.97 for naive Bayes classifier.^25^ Patil et al. predicted mortality at 0.978 for naive Bayes classifier, 0.961 for random forest, 0.961 for support-vector machine and 0.949 for back propagation.^26^

To the best of our knowledge, no previous publication has directly compared a set of different ML models with the commonly used Baux score and revised Baux score. Therefore, the aim of the present study was to evaluate a series of ML models to assess the level of accuracy that can be reached in outcome prediction for burn injuries, compared with two common prediction algorithms. This was a proof-of-concept study to explore different ML models for mortality prediction of intensive care burn patients and compare them to Baux score and revised Baux score.

## Methods

### Summary

**Figure 1.**
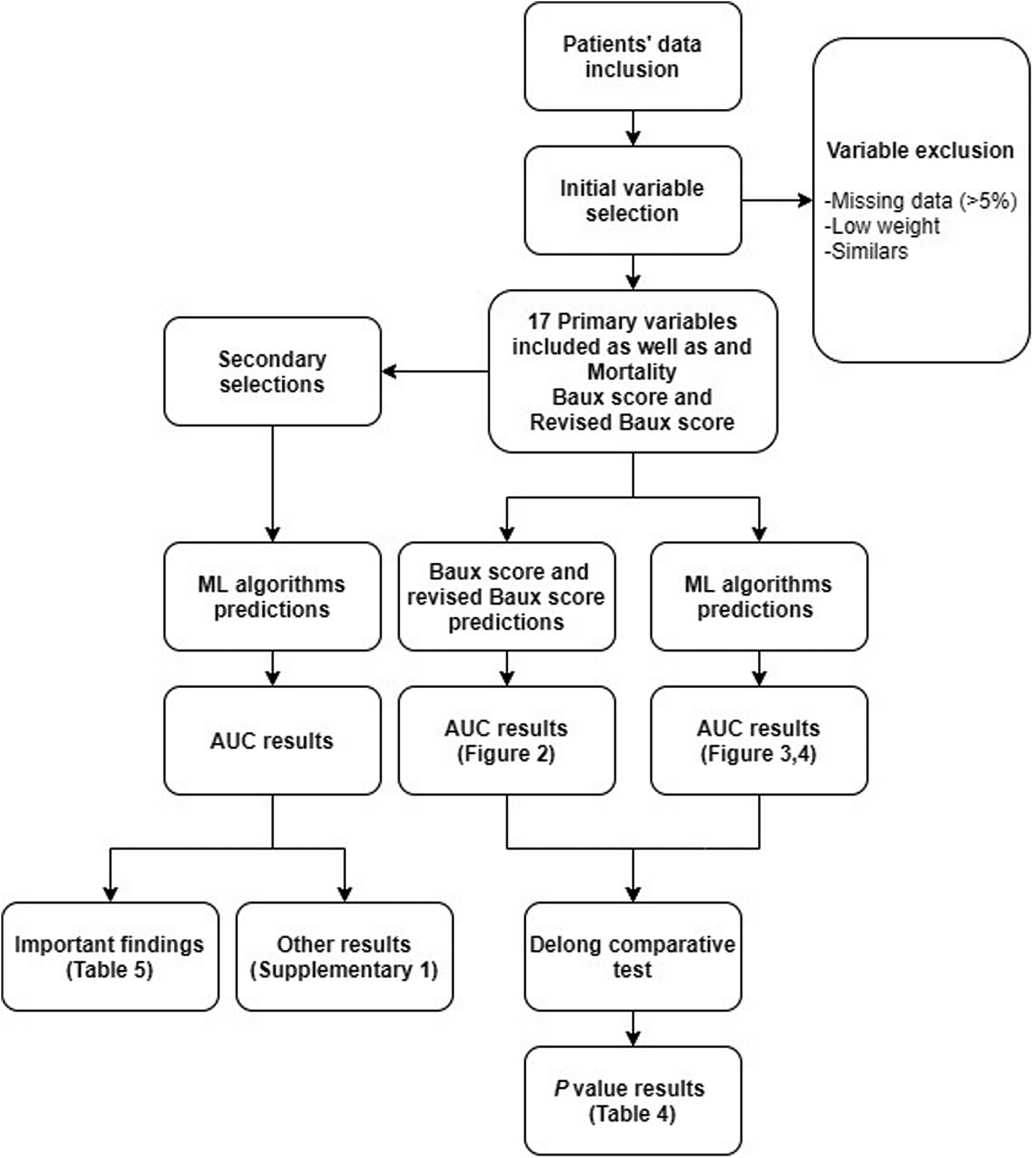
Flow chart of the data selection, modelling and evaluation of results.

Clinical and laboratory data, risk scores and mortality outcomes were gathered from each patient at admission. Selections of predictive variables were performed, and predictions were made through chosen ML algorithms compared to Baux score and revised Baux score. A summary of methods is depicted in Figure 1.

### Setting

Research was carried out at the Burn Centre in Uppsala University Hospital, one of two national burn centres in Sweden.

### Ethics review

The study is non-interventional and was approved by the local ethics review board in Uppsala, Sweden (Etikprövningsnämnden Uppsala, Dnr 2016/279). The procedure for consent was in line with recommendations by the board (International Conference on Harmonisation – Good Clinical Practice).

All patients with included data gave permission through an informed consent form sent by regular mail and returned a signed copy. If the patient was deceased, the informed consent form was sent to the patient's closest relative. The patient or relative was encouraged to contact the study investigators with additional questions and some patients or relatives had a phone call with a study investigator before giving written approval.

### Data gathering and selection

Only data on the day of the patient's admission were collected through the hospital's electronic health records (EHR) systems. Variables were divided into clinical data, laboratory data, compound risk scores, comparative risk scores (Baux and revised Baux score) and the outcome variable (mortality). The following EHR systems were accessed: Cosmic version R8.2.02 (Cambio Healthcare Systems AB, Stockholm, Sweden) and PASIVA version 4.3 (Otimo Data AB, Kalmar, Sweden), which covered patients admitted to the burn centre between 2002 and 2019. A non-randomised convenience sampling was exercised, which meant that patients who had sufficient data and provided informed consent first were included. No effort was made to assure that the cases were evenly distributed between groups regarding confounding factors, such as age or TBSA among others. Oversampling of deceased cases, with a target set at 30% of total data, was performed since most participants survived their injuries.

Variables with insufficient data (>5% of total data missing) were excluded. The outcome variable ‘deceased’ was defined as a patient who had died during the hospital stay at the burn centre or at the referring hospital within 10 days after return transfer, when the primary reason for admission had been a burn.

Risk scores were calculated using PASIVA internal algorithms. Separate recalculations were made on SAPS III scores using SAPS 3 research group calculation sheets to confirm validity.^29^

### Data preparation and variable filtering

Patients were pseudonymised and assigned a random study number for further analysis. Predictive variables were excluded based on the following criteria: missing data; variables deemed to have low impact on outcome; and variables that were similar to another variable that was already included. The latter two criteria were judged by two burn experts based on clinical experience.

### Software for calculations

Calculations were made using freeware Rstudio version 1.2.5033 and R data Miner Rattle version 5.3.0 (both Free Software Foundation, Inc., Boston, MA, USA). Scripts were prepared using R-packages Caret,^30^ Mleval^31^ and pROC.^32^

### Modelling, evaluation and comparison

ML algorithms for outcome prediction were selected based on their ability to accommodate multiple categorical and numerical input variables and produce a classification as output. The following algorithms were selected: decision tree; extreme boosting; random forest; support-vector machine (SVM); and a generalised linear regression model (GLM).

Leave-one-out cross-validation was used for training and testing the algorithms. Results were plotted as ROC curves, and AUC and confidence interval (CI) were calculated.

For Baux score and revised Baux score accuracy calculations, a simple regression model in relation to mortality outcome on the same dataset were used. Similar to the ML algorithms, CI was calculated through leave-one-out cross-validation and results were plotted as ROC curves with AUC values. Comparison between AUC for ROC curves was done using the DeLong test and the *P* values reported were two-tailed.^33^

### Secondary variable selection

After initial modelling and AUC calculations on selected variables, secondary variable selections were conducted to examine which variable, or group of variables when excluded, would impact the AUC of the models. This selection was performed based on the clinical experience of two burn experts.

## Results

### Data inclusion

Data from 92 patients (25 deceased) were gathered from the hospital's EHR at the time of admission. Initially, multiple clinical, laboratory and compound risk score factors were considered. Due to lack of data (>5% missing), eight variables were excluded. Sex was considered to have a low correlation with outcome and was therefore excluded. Two variables were excluded due to similarities with included variables (Table 1).

**Table 1.** Initial variables gathered at patient admission.

Clinical	Heart rate (beats/min)	Risk scores
Age (years)	TBSA total (%)	SOFA*
Sex (Male/Female)^†^	TBSA superficial dermal (%)	Charlson Co-morbidity Index*
BMI (kg/m^2^)	TBSA mid dermal/indeterminate (%)	SAPS III
MAP ( mmHg)	TBSA deep dermal (%)	SAPS III EMR (%)^‡^
RLS	TBSA full thickness (%)	Comparative risk scores
Temperature (°C)*	Laboratorial	Baux score
PaO_2_/FiO_2_ (P/F ratio)*	B-thrombocyte (10^9/L)	Revised Baux score
Diuresis (mL/day)*	S/P-bilirubin (µmol/L)	Outcome variables
Ventilator (Yes/No)	S/P-creatinine (µmol/L)	Mortality during admission (Yes/No)
Dialysis (Yes/No)*	B-leukocytes (10^9/L)	
Inhalation injury (Yes/No)	P-CRP (mg/L)^‡^	
Respiratory rate (breaths/min)*	B-pH*	

*Excluded variable due to missing data.

†Excluded variable due to low weight/correlation.

‡Excluded variable due to similarities to an included variable.

After initial selection, 17 input variables (Table 2), the comparative variables (Baux score and revised Baux score) and the outcome variable (mortality) were included in the primary predictive calculations.

**Table 2.** Variables included in primary data for prediction models.

Input variables	
1. B-thrombocyte	238.5 ± 90.5
2. S/P-bilirubin	17.1 ± 13.3
3. S/P-creatinine	83.8 ± 29.6
4. B-leukocytes	15.2 ± 6.7
5. MAP (mmHg)	71.8 ± 20.9
6. RLS	1.5 ± 1.2
7. Heart rate (beats/min)	88.9 ± 21.2
8. TBSA total (%)	25.4 ± 20.1
9. TBSA superficial dermal (%)	3.6 ± 6.2
10. TBSA mid dermal/ indeterminate (%)	7.8 ± 14.7
11. TBSA deep dermal (%)	5.9 ± 12.8
12. TBSA full thickness (%)	8.0 ± 16.0
13. Age (years)	62.3 ± 17.3
14. BMI (kg/m^2^)	26.9 ± 5.4
15. SAPS III	49.8 ± 11.2
16. Ventilator	
Yes	68
No	24
17. Inhalation injury	
Yes	37
No	55
*Comparative risk scores*	
Baux score	88 ± 25
Revised Baux score	95 ± 27
*Outcome variable*	
Deceased	
Yes	25
No	67

Values are given as n or mean ± SD.

BMI, body mass index; CRP, C-reactive protein; MAP, mean arterial pressure; RLS, reaction level scale; TBSA, total body surface area.

### Primary predictions

**Figure 2.**
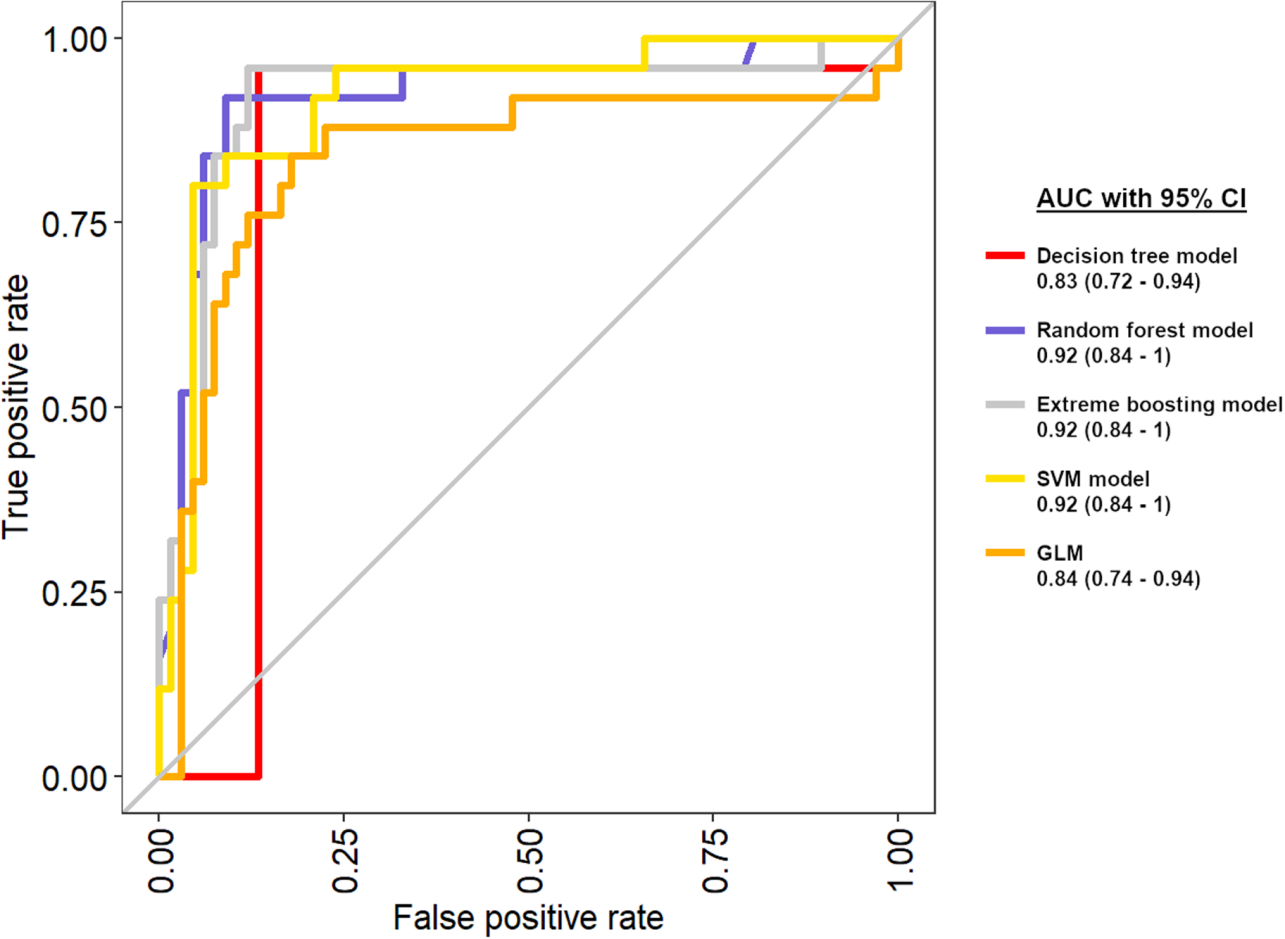
AUC after modelling and leave-one-out cross-validation: decision tree (red), random forest (blue), extreme boosting (grey), SVM (yellow), GLM (orange). Values in brackets are 95% confidence intervals. AUC, area under the curve; GLM, generalised linear regression model; SVM, support-vector machine.

AUC after leave-one-out cross-validation for the decision tree model, the extreme boosting model, the random forest model, the SVM model and the GLM were 0.83 (95% CI = 0.72–0.94) 0.92 (95% CI = 0.84–1.0), 0.92 (95% CI = 0.84–1.0), 0.92 (95% CI = 0.84–1.0) and 0.84 (95% CI = 0.74–0.94), respectively (Figure 2).

**Figure 3.**
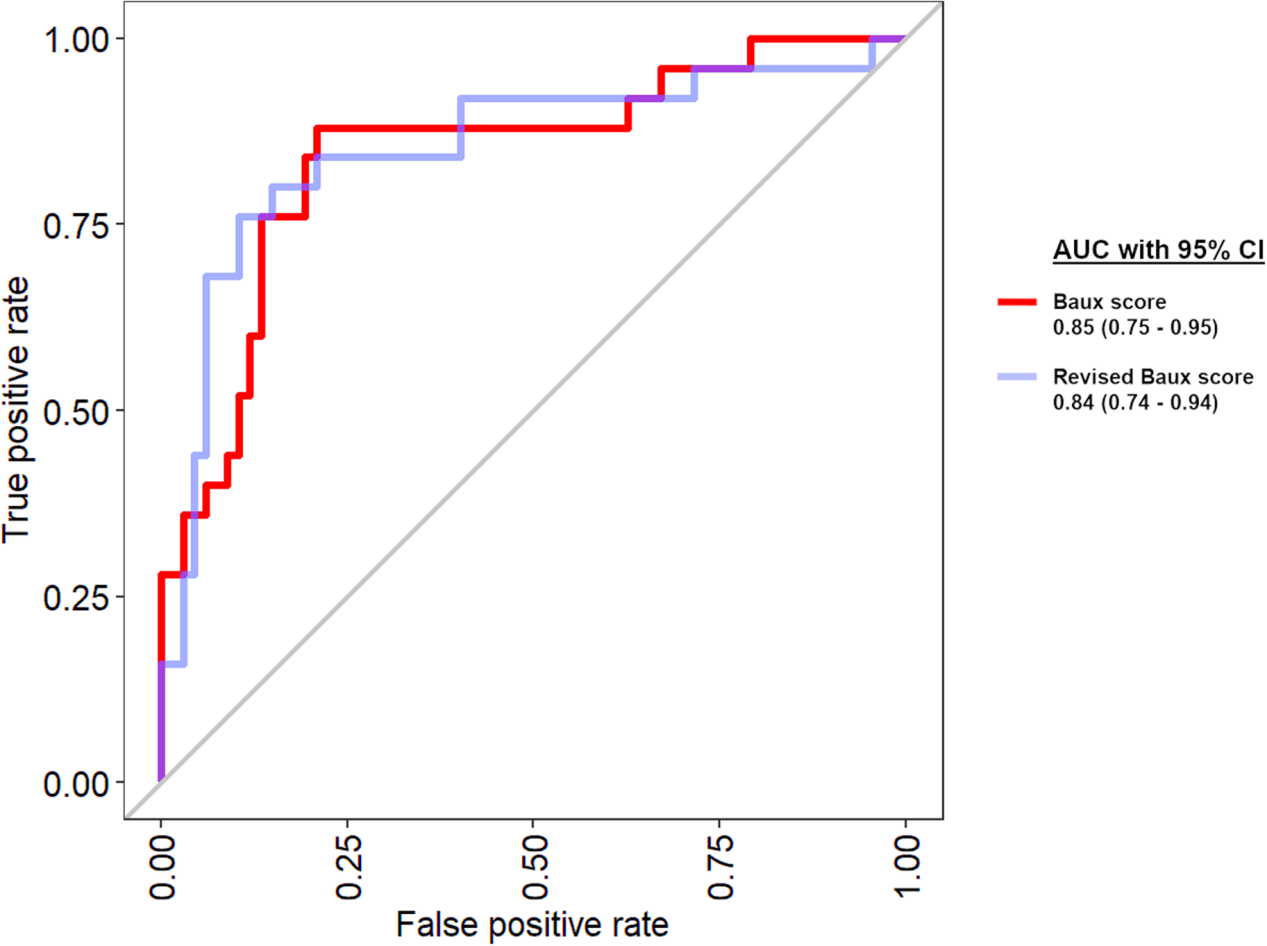
Area under the curve (AUC) after modelling for Baux score and revised Baux score. Values in brackets are 95% confidence intervals.

### Comparison of model performance

AUC results for Baux score and revised Baux score were 0.85 (95% CI = 0.75–0.95) and 0.84 (95% CI = 0.74–0.94), respectively.

*P* values, comparing ML models AUC with Baux score and revised Baux score, are shown in Table 3. There were no statistically significant differences observed (*P* < 0.05). Results for Baux score were the following: decision tree model (*P* = 0.55); extreme boosting model (*P* = 0.50); random forest model (*P* = 0.21); SVM model (*P* = 0.24); and GLM (*P* = 0.58). Results for the revised Baux score were as follow: decision tree model (*P* = 0.61); extreme boosting model (*P* = 0.33); random forest model (*P* = 0.10); SVM model (*P* = 0.14); and GLM (*P* = 0.70) (Figure 3).

**Table 3.** *P* values comparing AUC results of ML models with Baux score and revised Baux score.

	Baux score	Revised Baux score
Decision tree model	0.55	0.61
Extreme boosting model	0.50	0.32
Random forest model	0.21	0.10
SVM model	0.24	0.14
GLM	0.58	0.70

GLM, generalised linear regression model; SVM, support-vector machine.

### Prediction accuracy after secondary variable selection

AUC results of the assessed ML algorithms after secondary variable selection are presented in Table 4. Additional combinations examined are presented in Supplementary Material 1. Exclusion of SAPS III alone as well as in combination with extent of burn and age decreased the AUC. Exclusion of laboratory and clinical variables, not including extent of burn and age, showed minimal or no change in AUC. A flow chart of results is described in Supplementary Material 2.

**Table 4.** Prediction accuracy after secondary variable selection.

Variable(s) excluded	ML algorithm	AUC
SAPS III	Decision tree	0.78 (0.66–0.90)
	Extreme boost	0.83 (0.72–0.94)
	Random forest	0.87 (0.78–0.96)
	SVM	0.84 (0.74–0.94)
	GLM	0.76 (0.64–0.88)
Laboratory and clinical, not including burn extent and age (b)	Decision tree	0.83 (0.72–0.94)
	Extreme boost	0.92 (0.84–1)
	Random forest	0.92 (0.84–1)
	SVM	0.97 (0.92–1)
	GLM	0.89 (0.80–0.98)
Burn extent, SAPS III, age (c)	Decision tree	0.66 (0.53–0.79)
	Extreme boost	0.79 (0.68–0.90)
	Random forest	0.78 (0.66–0.90)
	SVM	0.78 (0.66–0.90)
	GLM	0.74 (0.62–0.86)

Values in parentheses are 95% CI.

AUC, area under the receiver operating characteristic curve; CI, confidence interval; GLM, generalised linear regression model; ML, machine learning; SVM, support-vector machine.

## Discussion

### Interpretation of results

This proof-of-concept study was conducted to assess a series of five ML algorithms in the prediction of short-term mortality due to a burn injury. Our exploratory results indicate that all ML algorithms and Baux scores through AUC-ROC curve analysis were sufficient in predicting mortality on the same dataset. When directly comparing the ML models with Baux score and revised Baux score, no statistically significant differences in performance were observed.

Revised Baux score has shown to be advantageous compared to Baux score in previous studies^5,8,34^ but no difference between the scores were observed in our study. Since the study was not designed to evaluate Baux scores, conclusions concerning the superiority of revised Baux score could not be drawn.

Breakdown of secondary variable selection showed that exclusion of laboratory data and clinical data, not related to burn extent and age, did not cause important changes in AUC. Prognostic laboratory markers for burn mortality have been studied,^35^ but no trends were observed in our study. Exclusion of SAPS III resulted in a reduced AUC. It is most likely that age and co-morbidities, which are included in SAPS III, have a high impact on mortality. Exclusion of age, extent of burn, and SAPS III presented even lower AUC. This is partially in line with variables that are included in the Baux score, which are deemed to be important predictive variables. However, results need to be interpreted with caution due to the risk of selection bias of variables. Therefore, further comparisons with Baux scores were deemed to be unwarranted.

### Limitations

Previous studies have shown that thousands of cases may be needed for machine learning approaches.^36–38^ Therefore, a larger sample size may benefit the training of ML algorithms in this study. Hence, the results presented in this paper could have been an effect of overfitting of data or chance and not generalisable to external, independent data. Therefore, the results need to be interpreted with caution. However, since all ML algorithms and Baux scores used the same dataset, a comparison between the traditional scores and ML models for a proof-of-concept study was deemed achievable.

Burn cases were selected based on convenience sampling that is described in the ‘Methods’ section. Hence no effort was made to assure even distribution regarding confounding factors. This approach may have skewed the type of patients included. Another aspect of the current study to consider is that an oversampling of deceased cases was performed. Therefore, the prediction accuracy of the models on unselected patients could be lower than the reported figures.

Every ICU has its intrinsic characteristics due to case mix, guidelines, resources and clinical traditions that affect outcome. The method presented in this study could be considered an example of a locally adapted ML model. The model, when used only locally, may have its advantages^39^ due to it being adapted to the conditions at hand. However, it can be argued that a multicentre trial would create algorithms that have a lower risk of overfitting and the algorithm trained can be generalized and be of further use in other burns ICUs beyond one's own hospital. Including data from other burn ICUs can also increase the amount of data available for training thus improving accuracy. The prerequisite in a multicentre trial would be that the ICUs should be similar in characteristics. If the characteristics are too diverse, you may run the risk of instead underfitting the data provided thus lowering accuracy of predictions.

In terms of variable selection, temperature and respiratory rate were excluded due to a lack of data, and C-reactive protein was excluded due to it being similar to leukocyte count as a biomarker for inflammation. Since these variables might be important predictors of sepsis and subsequently mortality rate,^40,41^ it can be speculated that the inclusion of these variables might have further optimised the accuracy of the ML models. In addition, diuresis and Charlson Co-morbidity Index, which can be necessary for clinical mortality prediction, were excluded due to a lack of data.

Variable selection is an intricate balance, where the inclusion of all clinically relevant variables may increase prediction accuracy. However, too many variables could cause overfitting, thus training the algorithms only to be accurate for the specific data gathered. Furthermore, variables are often causally related in clinical practice, and overlapping variables could create redundancies, thus slowing down algorithm performance.^42–44^

In this study, we considered the balance described above during variable inclusion. Nonetheless, practical limitations during data gathering may have impacted the results. However, since the ML algorithms and Baux scores all used the same variables for modelling, the results from the comparison between ML models and traditional scores may still add value.

## Conclusion

A proof-of-concept study on the prediction of mortality in burn intensive care patients using ML algorithms was on par with the Baux score and the revised Baux score. The study showed initial feasibility of the methods but, due to several limitations, need to be interpreted with caution. Further studies will require larger sample sizes as well as in-depth analyses of variable inclusion and exclusion. It also might be crucial to include external validation on independent data, prospective testing and evaluation of the models in real-world clinical decision making.

## Supplemental Material

sj-docx-2-sbh-10.1177_20595131211066585 - Supplemental material for A proof-of-concept study on mortality prediction with machine learning algorithms using burn intensive care data Click here for additional data file. Supplemental material, sj-docx-2-sbh-10.1177_20595131211066585 for A proof-of-concept study on mortality prediction with machine learning algorithms using burn intensive care data by Jian Fransén, Johan Lundin, Filip Fredén and Fredrik Huss in Scars, Burns & Healing

sj-jpg-3-sbh-10.1177_20595131211066585 - Supplemental material for A proof-of-concept study on mortality prediction with machine learning algorithms using burn intensive care data Click here for additional data file. Supplemental material, sj-jpg-3-sbh-10.1177_20595131211066585 for A proof-of-concept study on mortality prediction with machine learning algorithms using burn intensive care data by Jian Fransén, Johan Lundin, Filip Fredén and Fredrik Huss in Scars, Burns & Healing
