# Supplementary material for: A proof-of-concept study on mortality prediction with machine learning algorithms using burn intensive care data
Source: Scars Burn Heal. 2022 Feb 18;8:20595131211066585. doi: 10.1177/20595131211066585 (PMC8859689; doi:10.1177/20595131211066585)
Supplement: sj-docx-2-sbh-10.1177_20595131211066585 - Supplemental material for A proof-of-concept study on mortality prediction with machine learning algorithms using burn intensive care data [file sj-docx-2-sbh-10.1177_20595131211066585.docx]

Supplementary 1. AUC results by selected ML algorithms and secondary exclusion of variables in prediction of patient outcome (mortality). Numbers in brackets are representing 95% CI. Variable excluded is assigned a number and specified in Table 2.

| Variable(s) excluded | Decision Tree | Extreme Boost | Random Forest | SVM | GLM |
| --- | --- | --- | --- | --- | --- |
| 1 | 0.83 (0.72-0.94) | 0.91 (0.83-0.99) | 0.92 (0.84-1) | 0.92 (0.84-1) | 0.84 (0.74-0.94) |
| 2 | 0.83 (0.72-0.94) | 0.92 (0.84-1) | 0.92 (0.84-1) | 0.93 (0.86-1) | 0.85 (0.75-0.95) |
| 3 | 0.83 (0.72-0.94) | 0.92 (0.84-1) | 0.92 (0.84-1) | 0.93 (0.86-1) | 0.85 (0.75-0.95) |
| 4 | 0.83 (0.72-0.94) | 0.92 (0.84-1) | 0.92 (0.84-1) | 0.91 (0.83-1) | 0.86 (0.76-0.96) |
| 1, 2 | 0.83 (0.72-0.94) | 0.92 (0.84-1) | 0.93 (0.86-1) | 0.93 (0.86-1) | 0.85 (0.75-0.95) |
| 1, 3 | 0.83 (0.72-0.94) | 0.94 (0.87-1) | 0.92 (0.84-1) | 0.93 (0.86-1) | 0.85 (0.75-0.95) |
| 1, 4 | 0.83 (0.72-0.94) | 0.93 (0.86-1) | 0.93 (0.86-1) | 0.94 (0.87-1) | 0.86 (0.76-0.96) |
| 2, 3 | 0.83 (0.72-0.94) | 0.92 (0.84-1) | 0.91 (0.83-0.99) | 0.94 (0.87-1) | 0.86 (0.76-0.96) |
| 2, 4 | 0.83 (0.72-0.94) | 0.92 (0.84-1) | 0.92 (0.84-1) | 0.93 (0.86-1) | 0.87 (0.78-0.96) |
| 3, 4 | 0.83 (0.72-0.94) | 0.93 (0.86-1) | 0.93 (0.86-1) | 0.92 (0.84-1) | 0.87 (0.78-0.96) |
| 1,2,3 | 0.83 (0.72-0.94) | 0.92 (0.84-1) | 0.92 (0.84-1) | 0.94 (0.87-1) | 0.86 (0.76-0.96) |
| 1, 3, 4 | 0.83 (0.72-0.94) | 0.92 (0.84-1) | 0.93 (0.86-1) | 0.94 (0.87-1) | 0.87 (0.78-0.96) |
| 1,2,4 | 0.83 (0.72-0.94) | 0.91 (0.83-0.99) | 0.93 (0.86-1) | 0.94 (0.87-1) | 0.86 (0.76-1) |
| 2, 3, 4 | 0.83 (0.72-0.94) | 0.90 (0.82-0.98) | 0.92 (0.84-1) | 0.93 (0.86-1) | 0.88 (0.79-0.97) |
| 1- 4 | 0.83 (0.72-0.94) | 0.92 (0.84-1) | 0.92 (0.84-1) | 0.96 (0.91-1) | 0.87 (0.78-0.96) |
| 5 | 0.83 (0.72-0.94) | 0.92 (0.84-1) | 0.93 (0.86-1) | 0.92 (0.84-1) | 0.84 (0.74-0.94) |
| 6 | 0.83 (0.72-0.94) | 0.92 (0.84-1) | 0.92 (0.84-1) | 0.91 (0.83-0.99) | 0.84 (0.74-0.94) |
| 7 | 0.83 (0.72-0.94) | 0.92 (0.84-1) | 0.93 (0.86-1) | 0.92 (0.84-1) | 0.84 (0.74-0.94) |
| 5, 6 | 0.83 (0.72-0.94) | 0.93 (0.86-1) | 0.91 (0.83-0.99) | 0.92 (0.84-1) | 0.84 (0.74-0.94) |
| 5, 7 | 0.83 (0.72-0.94) | 0.92 (0.84-1) | 0.92 (0.84-1) | 0.92 (0.84-1) | 0.85 (0.75-0.95) |
| 6, 7 | 0.83 (0.72-0.94) | 0.92 (0.84-1) | 0.91 (0.83-0.99) | 0.94 (0.87-1) | 0.85 (0.75-0.95) |
| 1-4, 5-7 (b) | 0.83 (0.72-0.94) | 0.92 (0.84-1) | 0.92 (0.84-1) | 0.97 (0.92-1) | 0.89 (0.80-0.98) |
| 5-7 | 0.83 (0.72-0.94) | 0.91 (0.83-0.99) | 0.92 (0.84-1) | 0.94 (0.87-1) | 0.86 (0.76-0.96) |
| 8 | 0.83 (0.72-0.94) | 0.91 (0.83-0.99) | 0.93 (0.86-1) | 0.92 (0.84-1) | 0.84 (0.74-0.94) |
| 9 | 0.83 (0.72-0.94) | 0.93 (0.86-1) | 0.92 (0.84-1) | 0.92 (0.84-1) | 0.84 (0.74-0.94) |
| 10 | 0.83 (0.72-0.94) | 0.92 (0.84-1) | 0.92 (0.84-1) | 0.91 (0.83-0.99) | 0.84 (0.74-0.94) |
| 11 | 0.83 (0.72-0.94) | 0.92 (0.84-1) | 0.91 (0.83-0.99) | 0.94 (0.87-1) | 0.84 (0.74-0.94) |
| 12 | 0.83 (0.72-0.94) | 0.93 (0.86-1) | 0.93 (0.86-1) | 0.93 (0.86-1) | 0.84 (0.74-0.94) |
| 8-11 | 0.83 (0.72-0.94) | 0.93 (0.83-0.99) | 0.93 (0.86-1) | 0.91 (0.83-0.99) | 0.86 (0.76-0.96) |
| 8-10 | 0.83 (0.72-0.94) | 0.92 (0.84-1) | 0.93 (0.86-1) | 0.91 (0.83-0.99) | 0.86 (0.76-0.96) |
| 9,10 | 0.83 (0.72-0.94) | 0.92 (0.84-1) | 0.92 (0.84-1) | 0.92 (0.84-1) | 0.86 (0.76-0.96) |
| 9-11 | 0.83 (0.72-0.94) | 0.92 (0.84-1) | 0.92 (0.84-1) | 0.92 (0.84-1) | 0.86 (.0.76-0.96) |
| 8-12 | 0.83 (0.72-0.94) | 0.91 (0.83-0.99) | 0.92 (0.84-1) | 0.94 (0.87-1) | 0.88 (0.79-0.97) |
| 9-12 | 0.83 (0.72-0.94) | 0.93 (0.86-1) | 0.92 (0.84-1) | 0.94 (0.87-1) | 0.86 (0.76-0.96) |
| 11, 12 | 0.83 (0.72-0.94) | 0.93 (0.86-1) | 0.93 (0.86-1) | 0.92 (0.84-1) | 0.85 (0.75-0.95) |
| 12 | 0.83 (0.72-0.94) | 0.92 (0.84-1) | 0.92 (0.84-1) | 0.92 (0.84-1) | 0.84 (0.74-0.94) |
| 8, 11, 12 | 0.83 (0.72-0.94) | 0.91 (0.83-0.99) | 0.92 (0.84-1) | 0.93 (0.86-1) | 0.87 (0.78-0.96) |
| 8, 12 | 0.83 (0.72-0.94) | 0.92 (0.84-1) | 0.93 (0.86-1) | 0.92 (0.84-1) | 0.85 (0.75-0.95) |
| 13 | 0.83 (0.72-0.94) | 0.92 (0.84-1) | 0.92 (0.84-1) | 0.92 (0.84-1) | 0.84 (0.74-0.94) |
| 15(a) | 0.78 (0.66-0.90) | 0.83 (0.72-0.94) | 0.87 (0.78-0.96) | 0.84 (0.74-0.94) | 0.76 (0.64-0.88) |
| 1-7, 9-12, 14-17 | 0.80 (0.69-0.91) | 0.85 (0.75-0.95) | 0.80 (0.69-0.91) | 0.82 (0.71-0.93) | 0.84 (0.74-0.94) |
| 1-7, 9-12, 14-16 | 0.80 (0.69-0.91) | 0.83 (0.72-0.94) | 0.80 (0.69-0.91) | 0.84 (0.74-0.94) | 0.83 (0.72-0.94) |
| 1-7, 14-17 | 0.71 (0.58-0.84) | 0.84 (0.74-0.94) | 0.85 (0.75-0.95) | 0.86 (0.76-0.96) | 0.83 (0.72-0.94) |
| 1-7, 14-16 | 0.71 (0.58-0.84) | 0.85 (0.75-0.95) | 0.84 (0.74-0.94) | 0.83 (0.72-0.94) | 0.82 (0.71-0.93) |
| 1-7, 14, 15, 17 | 0.71 (0.58-0.84) | 0.83 (0.72-0.94) | 0.86 (0.76-0.96) | 0.83 (0.72-0.94) | 0.81 (0.70-0.92) |
| 1-7, 14, 15 | 0.71 (0.58-0.84) | 0.84 (0.74-0.94) | 0.85 (0.75-0.95) | 0.85 (0.75-0.95) | 0.81 (0.70-0.92) |
| 1-7, 14, 16, 17 | 0.83 (0.72-0.94) | 0.91 (0.83-0.99) | 0.92 (0.84-1) | 0.94 (0.87-1) | 0.93 (0.86-1) |
| 1-7, 14, 16 | 0.83 (0.72-0.94) | 0.89 (0.80-0.98) | 0.92 (0.84-1) | 0.95 (0.89-1) | 0.92 (0.84-1) |
| 8-12, 13, 15 (c) | 0.66 (0.53-0.79) | 0.79 (0.68-0.90) | 0.78 (0.66-0.90) | 0.78 (0.66-0.90) | 0.74 (0.62-0.86) |
| 8-12, 13 | 0.83 (0.72-0.94) | 0.92 (0.84-1) | 0.92 (0.84-1) | 0.96 (0.91-1) | 0.89 (0.80-0.98) |
| 8-12, 13, 17 | 0.83 (0.72-0.94) | 0.91 (0.83-0.99) | 0.92 (0.84-1) | 0.95 (0.89-1) | 0.89 (0.80-0.98) |
| 8-12, 13, 15, 17 | 0.66 (0.53-0.79) | 0.79 (0.68-0.90) | 0.78 (0.66-0.90) | 0.78 (0.66-0.90) | 0.75 (0.63-0.87) |
| 8-12, 13, 15-17 | 0.66 (0.53-0.79) | 0.77 (0.65-0.89) | 0.78 (0.66-0.90) | 0.72 (0.59-0.85) | 0.74 (0.62-0.86) |
