# Supplementary figures and images for: A proof-of-concept study on mortality prediction with machine learning algorithms using burn intensive care data
Source: Scars Burn Heal. 2022 Feb 18;8:20595131211066585. doi: 10.1177/20595131211066585 (PMC8859689; doi:10.1177/20595131211066585)

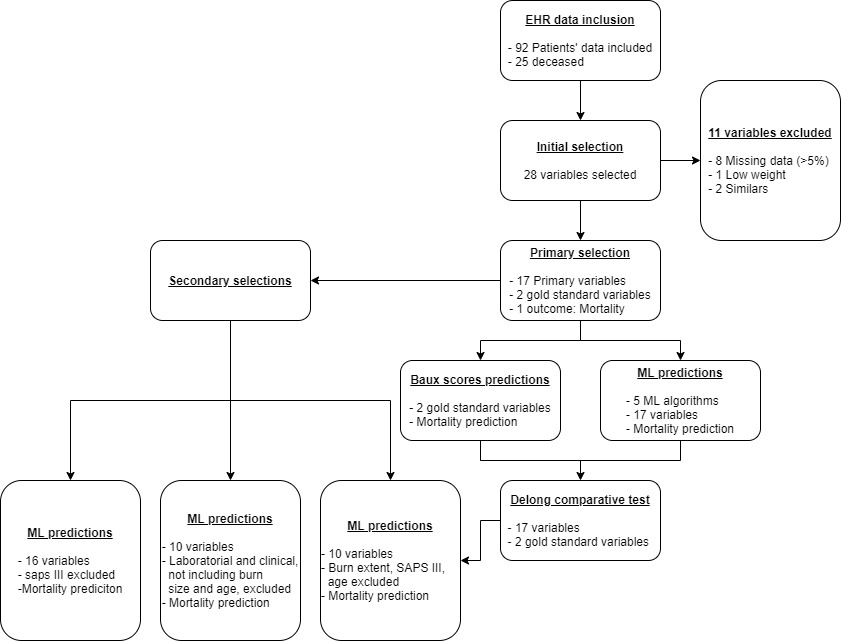

Supplement: sj-jpg-3-sbh-10.1177_20595131211066585 - Supplemental material for A proof-of-concept study on mortality prediction with machine learning algorithms using burn intensive care data [file sj-jpg-3-sbh-10.1177_20595131211066585.jpg]
